# Sensory–movement underpinnings of lifelong neurodivergence: getting a grip on autism

**DOI:** 10.3389/fnint.2025.1489322

**Published:** 2025-04-14

**Authors:** Steven K. Kapp

**Affiliations:** Centre for Interaction, Development and Diversity, School of Psychology, Sport and Health Sciences, Faculty of Science, University of Portsmouth, Portsmouth, United Kingdom

**Keywords:** autism, sensory, motor, development, regression, lost autism diagnosis, neurodiversity, support

## Abstract

While the autism diagnosis emphasizes “deficits” in social communication, the article advances that sensory–movement differences underpin autism through a review of the following sources of evidence. This account critically challenges “autistic regression”, with evidence that sensory–movement features appear by birth as the earliest signs of autism and underlie the behavioral differences used for diagnosis, which may reflect adaptations to inherent differences and misunderstandings from others. Sensory and motor differences are salient to autistic people, but they often go underrecognized by others. They cause cascading effects in infancy on behavior and communication through differences in sensorimotor learning, automatic imitation, eye contact, sensory perception, and interests. The article then explains how sensory processing differences may influence reduced perceptual narrowing, which involves a bottom-up information processing style grounded in the surrounding environment. Furthermore, this bottom-up processing may grow from reduced sensory integration in feedback loops potentially involving the cerebellum of the brain. The article then moves into implications for the widespread consequences of these inherent differences on quality of life. The article closes with implications for autism as a construct (including underestimated empathy and pain), testing the theory, providing sensory-sensitive support and acceptance of autistic people, and applications to diverse autistic people. The theory may apply particularly well to autistic women and girls, autistic people with speech divergence, autistic people with ADHD, and autistic people with co-occurring sensory and motor-related neurodivergences. Throughout the article, the theory also provides clinical, neurological, and experiential evidence for sensory and motor differences as lifelong, challenging the notion of “losing” (an) autism (diagnosis) as instead reflecting (risky and not necessarily “successful”) camouflaging.

## Introduction

Non-autistic people's definition of autism as based largely in social behavioral deficits within autistic people overlooks many things, including that neurological differences precede when people meet criteria (Dawson et al., 2023) and persist after some people learn to mask autism (Eigsti et al., 2016). The autism diagnosis is based on accounts by largely non-autistic people, yet only autistic people possess the expertise of direct lived experience, and many autistic people demonstrate enhanced scientifically based understanding of autism as well (Gillespie-Lynch et al., 2017). The diagnosis is defined by behavior, yet autistic people are more likely than non-autistic people to define autism internally (as related to cognition or biology: Gillespie-Lynch et al., 2017). It is defined pathologically on a deficit-only basis, whereas autistic people are more likely to define autism holistically (Gillespie-Lynch et al., 2017). Finally, the emphasis of diagnosis on social communication means it places social dysfunction within individuals, yet anything social happens *between* people (Kapp, 2013). Instead, the only measure of autistic traits developed in equal partnership with the autistic community recently reported extreme sensory processing and enjoyment of repeated actions as the only universally endorsed parts of autism (Ratto et al., 2023). Similarly, experiences of autistic adults have informed a view of autism as based in a “sensorium” affecting perception generally, with a drive toward relationships but the development of these is affected by others' misunderstanding of their needs (Murray et al., 2023; Milton and Green, 2024). This paper argues that neurologically based sensory–movement differences underpin autism from birth and remain for life, through mechanisms such as reduced perceptual narrowing of repeatedly encountered sensory experiences (Lewkowicz and Ghazanfar, 2009; Hadad and Yashar, 2022), which help explain why autistic people may find comfort in familiarity. This theory challenges notions that people “regress into” or “lose” autism, and has implications for supporting autistic people to leverage their differences and disabilities positively, as socially different rather than deficient.

## “Autistic regression” or autistic resilience? Coping with sensory–movement differences

Development is dynamic rather than continuous regression—a “loss of previously acquired skills not caused by brain injury or other traumatic events” (Zhang et al., 2019, p. 4)—is generally part of the human experience, even in infancy (Thelen and Spencer, 1998; Thelen, 2005; Brignell et al., 2017; Zwaigenbaum, 2019). For example, newborns generally have instincts for stepping motions that they seem to lose after a few months, explained because they cannot support the weight of their legs before they learn to walk (Thelen, 1995; Thelen and Fisher, 1982). Evidence and explanation for this come from Esther Thelen's dynamic systems theory (development as emergent and discontinuous in interaction with the environment: Spencer et al., 2006), from which Donnellan et al.'s (2010, 2013) sensory–movement account of autism borrowed. The latter defined autism as involving “sensory movement differences in starting, stopping, executing, combining, and/or switching actions, thoughts, emotions and speech” (Donnellan et al., 2010). It served as the only neurocognitive theory of autism in the first special issue on autism and the neurodiversity concept that featured an array of frontline activists and researchers (https://dsq-sds.org/index.php/dsq/issue/view/43). Inspired by “internal” components of evidence such as autistic people's lived experience and neuroscience, this account recognizes that behaviors may not reflect individuals' intent or may be misunderstood and seeks to practically apply ideas to accommodations for and supportive relationships with autistic people (Leary and Donnellan, 2012; Kapp, 2024). At the same time as recognizing the nuances of development, Donnellan (2008) arguably considered autism—or at least its underlying mechanisms—as present from birth, showing the coordination and timing needed for newborns to interact. Indeed, spontaneous movements in 2-day-old babies predict autism-related behavior in toddlers (Doi et al., 2023). Similarly to relatively recent evidence suggesting infants still have the stepping reflex but may not usually show it because of limited visual control (Barbu-Roth et al., 2015), even autistic infants or toddlers who seem to lose skills may have been autistic all along, especially as seen through differences in integrating sensory perception and movement.

The current empirically grounded framework of autistic people as—as Lady Gaga eloquently put it—“born this way” does not deny the appearance of (often temporary) developmental declines that many autistic people experience. Rather, it suggests that many of the behaviors that become more noticeable serve as coping mechanisms that show resilience in response to challenging (yet often vibrant) sensory inputs. It builds from long-standing evidence that sensory, motor, and general attentional differences (arguably themselves related to visual or eye-movement differences: Bellocchi et al., 2017) emerge as the earliest manifestations of autism (Gliga et al., 2014; Gallagher and Varga, 2015; Sacrey et al., 2015; Tanner and Dounavi, 2021), challenging “social-first” theories that posit differences in socially specific attention, motivation, or cognition cause autism (Rogers, 2009; Johnson, 2014; Falck-Ytter and Bussu, 2023). Indeed, autistic newborns do not show differences in social attention (Karmel et al., 2010; Cleary et al., 2023) but do show behavioral, physiological, and neurological differences related to sensation and movement if not also general attention such as hyperfocus (Cohen et al., 2013; Lucas and Cutler, 2015; Pineda et al., 2015; Miron et al., 2016; Ure et al., 2016; Liu et al., 2019; Cook et al., 2023; Doi et al., 2023; Tsang et al., 2024).

Rapin (1995) advanced the term “autistic regression” in the current sense, noting autism as reflecting differences that emerge over time, while notably singling out any poor motor skills as persistent. Some parents have instead conceived of “autistic regression” as meaning “typically developing” children suddenly acquired autism (Rapin, 1995), such as from environmental exposures like vaccines (Davidovitch et al., 2000). This constitutes one of many areas where parents and scientists have disagreements about the causation of and treatment for autism (Fischbach et al., 2016). Parents' narratives have borne little relationship to evidence from home videos, direct examination of the child in question, and prospective longitudinal research (Ozonoff et al., 2011; Pearson et al., 2018). Parents also often miss subtle early differences or delays (Ozonoff et al., 2010, 2015), which may include sensory and movement differences observable in home videos (Zappella et al., 2015; Paolucci et al., 2023). For example, autistic infants at 6 months may smile at least as much as non-autistic peers, but their smiling may be less contingent on the parents' communicative behaviors (Lambert-Brown et al., 2015). Similarly, early home videos suggest *enhanced* social responsiveness (Kaufman et al., 2022); overtly diminished early eye contact or responsiveness predicts *failing* to meet diagnostic criteria for autism (Clifford et al., 2013; Wagner et al., 2018). Instead, research suggests that most autistic people appear to show less normative behavior (e.g., less eye contact, gestures, or speech, or more social withdrawal or repetitive sensorimotor behaviors/“stimming”) in infancy through toddlerhood or early childhood, but that any changes happen gradually (Ozonoff et al., 2015; Bacon et al., 2018; Ozonoff and Iosif, 2019).

A history of early speech regression does not necessarily predict more challenges long term for autistic people than autistic peers without that history either. By middle childhood or at least young adulthood, autistic children with developmental regression histories often gain skills in understanding speech comparable to their autistic peers without that history (Pickles et al., 2022; Prescott and Ellis Weismer, 2022). Furthermore, over adolescence and young adulthood autistic people with a history of early speech regression demonstrated a *greater* increase in IQ in a population-based study (Simonoff et al., 2020). Nevertheless, while autistic people without intellectual disability but with early speech regression mostly attain fluent speech before adulthood (Gagnon et al., 2021), their production of speech may lag behind their understanding of speech, at least as older children (Pickles et al., 2022). This may hint at potentially greater apraxia of speech among some of them (oral-motor problems in speech production: Tierney et al., 2015; Vashdi et al., 2021; oral-motor problems occur in most autistic people: Maffei et al., 2023). Motor challenges like apraxia of speech may particularly affect non-speaking autistic people (Belmonte et al., 2013; Chenausky et al., 2019; Chen et al., 2024b; Maffei et al., 2024). Augmentative and alternative communication (AAC) systems such as speech-generating devices may help minimally speaking autistic people to gain speech and other communication (Kasari et al., 2014; Almirall et al., 2016; Logan et al., 2017), through their strong visual design and empowerment of fine motor control. Indeed, perceptual strengths in autistic people with speech-onset divergence may help them develop control over their vocal pitch (Sharda et al., 2010), arm (Barbeau et al., 2015; also see Fuentes et al., 2010), and eye (Takarae et al., 2004) movements, contributing to language and sometimes speech gains. They may sometimes also contribute to selective cognitive advantages over other autistic and non-autistic people Kapp and Gudknecht (Under review)^1^.

### Conceptual and empirical foundations of sensory–movement differences: the origins of autism

Sensory and movement differences appear to set autistic people on a divergent path by life's first moments. Piaget's 1930s seminal theory of cognitive development classified a child's first 2 years as the sensorimotor stage (Beilin and Fireman, 1999; Mostofsky and Ewen, 2011; see Bussu et al., 2021). Autistic infants appear to have relatively enhanced sensory if not movement skills followed by what observers commonly call stagnation or declines as autism becomes more apparent as toddlers (Nyström et al., 2018; Bussu et al., 2021; Fish et al., 2021; Tan et al., 2021; also see Wolff et al., 2012, 2015). While Piaget proposed needing mastery before being able to move on to the next stage, autistic people generally can learn new movements similarly as well as non-autistic people, but through different (less intuitive) mechanisms that require more focused attention (Mostofsky and Ewen, 2011; Lidstone and Mostofsky, 2021). Furthermore, autistic people with intellectual disabilities (e.g., associated with conditions linked to rare *de novo* genetic mutations) do tend to have more motor challenges than other autistic people and non-autistic people (Denisova, 2024). However, these motor differences transcend intellectual challenges (Buja et al., 2018), which may suggest that others may underestimate their cognitive capacities because of their movement-related behavioral or performance difficulties. Indeed, lacking speech for autistic toddlers may only challenge the ability to produce language (at least without access to AAC support), even in those with low cognitive performance (Delehanty et al., 2018). However, for peers with developmental disabilities, limited speech relates to worse visual cognition alongside difficulties producing *and* understanding language (Delehanty et al., 2018). Moreover, peers with and without developmental disabilities' but not autistic people's ability to understand language may draw from their visual perceptual skills (Delehanty et al., 2018; Hannant, 2018). Even autistic people with minimal expressive language tend to have autism-typical visual perceptual strengths, which may support their non-verbal cognition (Courchesne et al., 2015).

The earliest accounts of autism explicitly included sensory hypersensitivities (Sukhareva, 1925; Ssucharewa, 1927; Kanner, 1943; Asperger, 1944, 1991) and motor challenges (Sukhareva, 1925; Ssucharewa, 1927; Asperger, 1944, 1991), yet diagnostic manuals have usually and always omitted them respectively from autism's historical definitions (Rosen et al., 2021). The latest edition's addition of sensory features helped reduce false negatives without necessarily resulting in false positives (Frazier et al., 2012), suggesting their importance to the hallmark behaviors used for diagnosis. While the autism diagnosis, the phenomenon of regression, and indeed non-autistic people's definitions of autism tend to depend on observable behaviors or skills, autistic people tend to define autism as based on neurocognitive differences (Gillespie-Lynch et al., 2017). These include aspects historically excluded from the autism diagnosis, such as differences in sensory processing, information processing, and emotion regulation (Chamak et al., 2008). Sensory differences have long dominated autistic people's accounts and illustrate their widespread impact (Cesaroni and Garber, 1991; O'Neill and Jones, 1997; Jones et al., 2003; Keane, 2004; Davidson and Henderson, 2010). These narratives tend to emphasize hypersensitivity and difficulties with integration (e.g., simultaneously looking and listening: Robledo et al., 2012; Sibeoni et al., 2022), but they may manifest differently depending on variables such as energy and stress (Robertson and Simmons, 2015). Indeed, autistic people's sensory hyperreactivity and sometimes sensation seeking (which can relate to enhanced enjoyment related to hypersensitivity) show validity in psychometric measurement, unlike hyporeactivity (Williams et al., 2023). The same individual may show heightened or reduced biophysiological responses to the same stimuli, possibly reflecting awareness and ability to tune out aversive sensations (Khan et al., 2015a; Failla et al., 2018) or shutdown due to stress related to sensory overload (Mazefsky et al., 2014; Phung et al., 2021). Nevertheless, lacking the ability to filter out background noise may leave an autistic person unable to understand speech (Schwartz et al., 2020). This may necessitate a lack of distractions or presentation of language or symbols in other modalities (e.g., words on screen or printed: Ostrolenk et al., 2017). Indeed, among sensory modalities the auditory domain may most interrelate with autistic traits, although others (such as the more embodied tactile domain and sense of one's position) also prove important to social communication (Bang and Igelström, 2023).

Hypersensitive autistic people (including those with intellectual disability: Elise et al., 2025) may experience the ability to perceive higher perceptual loads of visual and auditory stimuli (Foss-Feig et al., 2013; Manning et al., 2015; Brinkert and Remington, 2020). This shows evidence in primary sensory cortices (e.g., visual cortex), related to more autistic traits (Ohta et al., 2012). This enhanced perceptual capacity can have benefits but may feel overwhelming (Irvine et al., 2024), in part, because autistic people may also notice more irrelevant stimuli (Tyndall et al., 2018).

Motor differences arguably often form the output to sensory input (especially for more movement-related senses such as vestibular [balance/movement] and proprioceptive [body position]: Ornitz, 1974; Gowen and Hamilton, 2013; Torres et al., 2013a). Indeed—like sensory processing—autistic people describe within-person variability in motor functioning (Gowen et al., 2023). They have detailed how motor difficulties pervasively affect life and require conscious attention or effort (Gowen et al., 2023). Approximately 90% of autistic people may have functionally impactful movement difficulties (Zampella et al., 2021; Bhat, 2023). Autistic people overlap with (Miller et al., 2024) and often struggle more than people with a neurodivergence defined by them (developmental coordination disorder/dyspraxia). They may have more significant differences in early motor development than dyspraxic peers (Bowler et al., 2024) and more difficulties with some movement-based skills such as gestures (Abrams et al., 2024). Compared with dyspraxic peers, autistic children and adolescents display lower motor anticipation (Martel et al., 2024) and body awareness (Hannant et al., 2018) alongside greater sensory hyper-reactivity (Hannant et al., 2018; Harrison et al., 2021; Ringold et al., 2022). Hence, so many autistic people experience motor difficulties that autistic people without a dyspraxia diagnosis tend to experience as so motor challenges (Hannant et al., 2018) and autism as deeply (Cassidy et al., 2016) as those with one, even though abundant evidence suggests motor difficulties help account for autistic people's executive functioning and communication challenges (Bhat, 2023; Estrugo et al., 2024; Gu et al., 2024). Nevertheless, few autistic people receive a formal dyspraxia diagnosis nor support for motor challenges (Cassidy et al., 2016; Zampella et al., 2021; Bhat, 2024).

Furthermore, non-autistic people tend to promptly form negative first impressions of autistic people's movements (Plank et al., 2023). Non-autistic people (e.g., children) particularly may stigmatize averted eye contact even beyond stimming behaviors such as hand-flapping (Sargent and Jaswal, 2022). Autistic children showed enhanced motor and social synchrony (including engagement and social awareness) with one another compared with pairs of non-autistic children (Glass and Yuill, 2024), illustrating the importance of social context for movement and other autistic differences. Compared with autistic peers, non-autistic people may require higher levels of social motor synchrony (Efthimiou et al., 2025) and their own social understanding (Morrison et al., 2020) for interactions with autistic partners to go well, which adversely affects autistic people (Kapp, 2018b; Caron et al., 2022).

In response to inherent differences and social experiences, autistic people develop so-called restrictive and repetitive behaviors and interests (RRBIs) that—according to their own reports and other evidence—help them navigate an often unpredictable, overwhelming world (Wigham et al., 2015). They help provide calming self-regulation and a sense of flow through engaging in interests and pleasurable activities (Collis et al., 2024; Long et al., 2024; Lung et al., 2024).

### Sensory and movement differences as underpinning diagnostic criteria for autism and remain across the lifespan

According to an emerging consensus based on empirical evidence, sensory–movement differences (e.g., visual perception as well as eye-movement and motor challenges) in infancy precede discernible communication differences associated with autism (Zhang et al., 2019). At 6 months, only reduced motor control and hand–eye integration distinguish autistic people from non-autistic infant siblings of autistic people, despite both sharing motor differences or delays, and both having more motor challenges than non-autistic peers (LeBarton and Landa, 2019; Zwaigenbaum et al., 2021). Lower hand–eye coordination at 12 months predicts the social communication of autistic toddlers via sensory differences (Capelli et al., 2024). Adding sensory features to autism assessment in infancy helped enable it predict autism earlier than ever (from 6 months: Zwaigenbaum et al., 2021). Similarly, the addition of sensory regulatory behavior to screening among children who meet preliminary criteria for autism at 12 months predicts more specific identification (fewer early false positives) of autism in children at the age of 30 months. It identifies children with greater and more persistent developmental difficulties, particularly in social communication (Ben-Sasson and Carter, 2013). Indeed, fine motor skills (Sutera et al., 2007) and *lack* of hyper-reactivity to (Troyb et al., 2014) and perception of (see Eigsti and Fein, 2013) sounds as toddlers predicted autistic children appearing non-autistic. Suggesting their cascading effects, these reduced sensorimotor differences in young autistic children were associated with fewer concurrent differences in repetitive behavior (Troyb et al., 2014) and communication (Fein et al., 2013). Similarly, reductions in stimming during toddlerhood predicted autistic people no longer meeting current behavioral diagnostic criteria in young adulthood (Anderson et al., 2014). Conversely, more significant motor challenges as toddlers predicted a major autism screener falsely missing autistic children (Shuster et al., 2024). Greater stimming at 12 months particularly predicts autism (Elison et al., 2014). In autistic toddlers stimming best predicted social communication differences, although all repetitive behaviors contributed to all the social interaction subdomains (Chaxiong et al., 2022).

Sensory sensitivities reduce tolerance of uncertainty in autistic people (see “Reduced perceptual narrowing”), helping explain soothing repetitive behaviors and anxiety (Wigham et al., 2015; Neil et al., 2016; Hwang et al., 2020; Powell et al., 2025). Greater behavioral reductions in social communication differences than in repetitive behaviors for autistic children and adults (Kapp, 2016; Waizbard-Bartov and Miller, 2023) hint further at the foundation and persistence of sensory differences. Autistic people report enduring sensory challenges and that they intensify by older adulthood (Chen et al., 2024a). Similarly, motor difficulties may hardly improve with age except fine motor skills in girls without intellectual disability (Biscaldi et al., 2014; Bhat, 2023). From middle age autistic people's involuntary movement differences tend to diverge further from non-autistic people's movements (Torres et al., 2020).

### Sensory and movement differences according to different reporters: often underestimated by non-autistic people

Autistic people's sensory and movement differences are difficult for other people to understand and may help explain why parents may perceive sudden “autistic regression”. Parental reports on their autistic infant's sensory responsiveness uniquely lacked reliability (Del Rosario et al., 2014). Similarly, parental reports on their autistic toddlers' sensory-related behaviors did not align with clinical observation (Ben-Sasson et al., 2007). Brain activity involving auditory, visual, and tactile processing shows a significant association with manifestations of autism or divergences from non-autistic peers even when parent reports or clinical measurements do not (Brandwein et al., 2015; Green et al., 2019). Autistic people's brain activity shows high within-person variability to the same stimuli in these domains (Haigh, 2018), and observers may struggle to interpret autistic people's inconsistent reactions (see Geurts et al., 2008). Fortunately, clinical interviews help parents recognize lifespan sensory differences they often miss (Leekam et al., 2007; Kent, 2014). Trends between parent and child reports on sensory processing suggest that parents may struggle to interpret sensory differences as their children become more aware of social reactions and begin to mask their differences. They show minimal agreement in their reports (MacLennan et al., 2020). Parents tend to report increasing sensory over-responsivity and activity until ages approximately 6 to 9 years and decrease thereafter through adulthood (Kern et al., 2006; Ben-Sasson et al., 2009). However, autistic boys without intellectual disability ages 6 to 18 years reported greater sensory difficulties with increasing age even as their mothers showed the opposite trend, with higher scores by self-report overall (Bitsika et al., 2016). Studies have consistently found the persistence of self-reported sensory sensitivities within adulthood (Crane et al., 2009; Kent, 2014). In contrast to parental reports of a relationship between sensory behaviors and autistic traits in children but not adolescents or adults (Kern et al., 2007), autistic adults have reported sensory and motor behaviors as most interrelated with the rest of autistic traits (Andersen et al., 2011). Similarly, autistic adults without intellectual disability report higher sensory differences and needs than caregivers or support staff of autistic people with intellectual disability report on their behalf (Bradshaw et al., 2024).

Autistic women's and girls' sensitivities may be particularly underrecognized, in part due to greater camouflaging (Lai et al., 2011; Harstad et al., 2023; see Pagán et al., 2024; Taylor et al., 2024). Autistic females have greater sensory sensitivities than autistic males (Kumazaki et al., 2015; Lai et al., 2011; Osório et al., 2021; Kose et al., 2025; Saure et al., 2023), whereas relatively greater motor skills over time (Bhat, 2023) may enable them to mask their autism. Sensory processing difficulties may grow in adulthood, especially for autistic women (Chen et al., 2024a), contributing to distress and chronic illness with increasing age (Grant et al., 2022, 2025; Chen et al., 2024a). They appear to be a primary driver of an autism diagnosis, especially for females (Fry, 2024). However, white, non-Hispanic males with highly educated mothers are disproportionately represented among those with clinician-documented sensory features (Kirby et al., 2022).

### Cascading impacts of sensory and movement differences in infancy

All behavior at a basic level stems from movement (Adolph and Berger, 2015), while motor delays tend to grow for autistic people over infancy (West, 2019; Lim et al., 2021). The following marked the earliest predictors of autism in studies that followed up over multiple time points: (a) atypical visual tracking alongside weak arm motor tone at 1 month (Karmel et al., 2010), (b) less advanced visual reception and gross motor skills at 6 months (Estes et al., 2015), and (c) little grabbing at toys or throwing them to the floor in addition to lacking the postural ability to sit on a lap with a straight back also at 6 months (Lemcke et al., 2013). These provide evidence of divergent early perception and action that constrains the ability to explore the environment within the first 6 months of infants later diagnosed with autism: hypotonia (Samango-Sprouse et al., 2015), lack of oral-motor anticipation (Brisson et al., 2012) or ability to stop sucking without support (Lucas and Cutler, 2015), less symmetrical and more basic postures (Esposito et al., 2009), delays in the development and spontaneous initiation of new postures (Nickel et al., 2013), head lags when pulled to sit (Flanagan et al., 2012; also see Bradshaw et al., 2023), greater observation of but less oral and manual engagement with objects (Kaur et al., 2015; Focaroli et al., 2024), increased perceptual sensitivity (Clifford et al., 2013), shorter spontaneous visual fixations to static stimuli (Wass et al., 2015), suspected vision problems such as squinting (Bolton et al., 2012), and atypical general movements (Zappella et al., 2015). They may manifest as less “liveliness” and subtler bids for attention (Wan et al., 2013), and passivity with decreased activity and intensity (Zwaigenbaum et al., 2005; Bolton et al., 2012; Del Rosario et al., 2014).

Infants may already manifest early sensorimotor development of autism (enhanced perception and movement challenges) even before diagnostic instruments recognize them as autistic, through a lack of flexible face scanning (see “Eye contact”) and little motor initiation (also see Bryson et al., 2007). This includes low activity and high perceptual sensitivity at the time infants typically begin to vocalize but nevertheless exhibit average to high social responsiveness in terms of attention to faces of their mothers (Rozga et al., 2011) or examiners (Ozonoff et al., 2010); smiling, cooing, and laughing (Clifford et al., 2013; Del Rosario et al., 2014) alongside enhanced perceptual sensitivity (Clifford et al., 2013); atypical general movements (Zappella et al., 2015); and low activity (Del Rosario et al., 2014). The following may indicate a self-regulatory function of stimming in individuals too sensitive for soothing from touch and who lacked the motor coordination to self-produce the rhythmic movements in early infancy: (a) low activity in socially responsive infants at 6 months that precedes high activity and autism diagnosis at 2 years (Bolton et al., 2012; Del Rosario et al., 2014), and (b) high perceptual sensitivity and apparently typical social responsiveness in infants at 6 months that precedes reduced pleasure from rocking by parents and more negative affect driven by less “cuddliness” in 2-year-old autistic toddlers (Clifford et al., 2013). Indeed, poorer sensory–movement skills in infancy predict autism via difficulties with self-regulation of attention and emotion (Perry et al., 2024). Focused repetitive movements and behaviors may help to self-regulate attention and emotion (Kapp et al., 2019b; see McKinnon et al., 2019). For example, an autistic infant experienced problems with oral motor coordination and muscle tone in the first 6 months of life, and his pattern of tactile and startle sensitivity alongside poor self-regulation predicted problems in social interaction despite early smiling and cooing (Dawson et al., 2000). Similarly, infants sleeping *more* at 5 months and *less* at 14 months predicted a diagnosis of autism (Begum-Ali et al., 2023), as the infants became more physically independent and potentially experienced more sensory overload (see “Reduced perceptual narrowing”).

#### Sensorimotor learning

Autistic people demonstrate lifelong slower movement and related executive functioning that contribute to their manifestations of autism (Cho et al., 2022; Jertberg R. et al., 2024; Wilson et al., 2024). Autistic children's differences in action planning and control become greater with age (Chua et al., 2022). Autistic children's dyspraxia entails poor manual dexterity to command (e.g., gesture), use tools (e.g., control and share objects as in playing), and consciously imitate (Dziuk et al., 2007). These difficulties distinguish autistic children from the motor difficulties of attention-deficit hyperactivity disorder (ADHD; MacNeil and Mostofsky, 2012) and even developmental coordination disorder (Kilroy et al., 2022b). Indeed, imitation abilities reflect not only motor skills in autistic children (Vanvuchelen et al., 2007) but also a closer interrelationship between motor and imitation skills than in ADHDers and comparison groups (Biscaldi et al., 2015). Significant challenges in imitating facial movements and hand and finger gestures improve somewhat over time (Biscaldi et al., 2014) but even speaking adults tend to have less accurate imitation and rely on goals to consciously emulate (Wild et al., 2012; see Hamilton, 2008; Edwards, 2014), while robust difficulties with timing of motor performance and movement quality remain (Biscaldi et al., 2014).

Autistic people may learn to move by focusing on their own body both during infancy (e.g., looking at one's lower body and the floor when learning to walk) and well after infancy. Despite early motor delays, autistic people often fare relatively well in learning to walk (Bowler et al., 2024; Wilson et al., 2024) compared with others with significant developmental disabilities (Bishop et al., 2016) and other motor difficulties (Ming et al., 2007). This may occur in part because of attention to their proprioception. For example, autistic infants may shift their attention from eye contact (Jones and Klin, 2013; Rutherford et al., 2015) to their movement and the floor to learn to crawl (see Kretch et al., 2014). A pattern of motor learning and control typical of autistic children involves visual attention to their body's position in space rather than looking outward (Masterton and Biederman, 1983; Morris et al., 2015; Sharer et al., 2016), related to difficulties with imitation and general manifestations of autism (Haswell et al., 2009). This may form an adaptation to differences in visual–motor integration (Linkenauger et al., 2012; Nebel et al., 2016; Lidstone and Mostofsky, 2021) and postural stability (Travers et al., 2013; Lim et al., 2017; Oster and Zhou, 2022) that also relate to challenges participating in many interactions. Others may misperceive why autistic people may need to actively focus on their bodies, as clinicians rated autistic children who had a more accurate sense of body ownership as displaying less empathy (Cascio et al., 2012). Autistic people's sense of their body may require conscious attention, as they may not automatically incorporate proprioceptive feedback (Shafer et al., 2021).

Indeed, a micro-movement model aligned with the sensory–movement theory has found evidence of more variable and random-looking signatures of fluctuations in pointing movements, reflecting a lack of automatic proprioception among autistic people. It contrasts with typical development by preschool age of intuitive proprioception built from experience that enables flexible, timely, and spontaneous gestures (Brincker and Torres, 2013; Torres et al., 2013a,b; also see Simeoli et al., 2019). Gestures that point at objects or hold them help build vocabulary in both autistic and non-autistic children (Özçalişkan et al., 2016). However, gestural divergence uniquely distinguished autistic children from language-impaired and developmentally disabled peers in infancy and toddlerhood (Veness et al., 2012; West et al., 2024). This may reflect weaker manual dexterity in autistic children than in children with language disability even though they experience some motor challenges (McPhillips et al., 2014).

Motor skills help autistic children with minimal speech understand speech and language. They may be the largest contributor to a gap between (higher) understanding than expression of language (Chen et al., 2024b). However, motor problems contributing to a lack of gestures or pointing may also lead to an underestimation of these children's intellectual and communicative abilities (Krueger, 2013; Courchesne et al., 2015; Girard et al., 2022; Kapp, 2023). For example, the children may make clear but unreciprocated bids for communication (Krueger, 2013). Fine motor skills play a role in common routes to joint engagement (Bhat et al., 2011; Jaswal and Akhtar, 2019), which promotes language development with a supportive partner (Adamson et al., 2019). In minimally speaking autistic preschoolers, fine motor skills most strongly predicted expressive language in young adulthood (Bal et al., 2020).

Grasping difficulties may affect speech or communication development. For example, limited fine motor skills contributed to autistic children's lack of (neuro)typical shape bias for word learning (Potrzeba et al., 2015), a unique finding that may occur because of difficulties grasping objects or gesturing. Furthermore, weak handgrip is associated with more obvious communication differences in autistic people across age, regardless of history of “regression”, and across IQ (Kern et al., 2011; Travers et al., 2015). Autistic people tend to have difficulty executing reaching and grasping actions that transact with others' responses to cascade into affecting interactions (Sacrey et al., 2014b). In contrast to gross motor skills' relationship to general language (including understanding) and communication in autistic people (Bedford et al., 2016; Hannant, 2018), poor oral-motor and fine motor skills in people in infancy through early childhood particularly relate to speech production (Iverson and Wozniak, 2007; Gernsbacher et al., 2008a; Belmonte et al., 2013; LeBarton and Iverson, 2013). This is especially the case among autistic people with minimal speech (Chenausky et al., 2019; Butler and Tager-Flusberg, 2023).

#### Echopraxia and echolalia: Automatic imitation

Autistic people often struggle with volitional but usually not automatic actions, such as eye movements (Minshew et al., 1999), gaze following (Kirchgessner et al., 2015), bimanual lifting (Martineau et al., 2004), locomotion, balance control (Vernazza-Martin et al., 2005), and grip (Stoit et al., 2013). The ability to spontaneously and precisely match another's movements in young autistic children predicts spoken language growth (Stone and Yoder, 2001; Miniscalco et al., 2014).

The typical or high social responsiveness as conventionally understood in infants later diagnosed with autism may partially reflect automatic hyperimitation of others' actions, or *echopraxia*, in association with more obvious autism and developmental disability. In autistic adults (a) automatic hyperimitation of hands and (b) (on a “theory of mind” task) not pretending that triangles have mental states ironically relates to others' perception of them as lacking in social and emotional reciprocity (Spengler et al., 2010). Indeed, automatic hyperimitation in autistic people especially applies to a greater bias toward copying human over robot hand movements (Bird et al., 2007). Similarly, automatic hyperimitation of fingers relates to more obvious autism (Sowden et al., 2016). Other studies consistently find evidence of at least typical levels of automatic imitation in autistic people (Hamilton et al., 2007; Press et al., 2010; Schunke et al., 2016).

Autistic people's differences in self-other distinction in automatic imitation appear to be at a sensory-motor level (Cracco et al., 2018). Increased within-person sensorimotor variability in autistic individuals challenges higher level integration of information for efficient motor planning and control (Gowen and Hamilton, 2013; Torres et al., 2013a; Fourie et al., 2025). This may contribute to the reduced impact of experience on an implicit sense of one's own actions that most people rely on for quick perception of others' behaviors as in joint attention (c.f. Mundy et al., 2010; Mostofsky and Ewen, 2011; Donnellan et al., 2013). Autistic children of various abilities and support needs demonstrate high responsiveness to adults' imitation of their behavior (Nadel, 2015), as they may raise self- and other awareness and incorporate greater reciprocity from the other (Gernsbacher, 2006). Similarly, when parents synchronize or match their behavior (e.g., mirroring the child's rhythm or pace) in response to their autistic child's, this arguably reflects a form of the adults' imitation of the child, and predicts higher joint engagement and child language (Gulsrud et al., 2016).

Similarly, speech apparently lost may have been *echolalia* (Clarke, 2019): imitation of another's language (Jaswal and Akhtar, 2019). Speaking single words earlier but later development of more flexible and complex language may reflect echolalia and relates to autistic children appearing to regress (Gagnon et al., 2021). Higher echolalia in response to direct communication relates to poorer adaptive functioning among institutionalized autistic adults with low language (Grossi et al., 2013). Echolalia may help autistic and non-autistic people learn language (Haydock et al., 2024), and almost all autistic children and youth may engage in it (Maes et al., 2024). However, if communication partners regard it as a repetitive behavior (American Psychiatric Association, 2013) rather than a form of communication, they may miss opportunities to connect.

#### Eye contact

Autistic infants tend to at first make at least as much eye contact as non-autistic peers (Falck-Ytter, 2024); challenges at the oculomotor (eye movement), auditory filtering, and oral motor levels for sensory integration appear most relevant for declines in eye contact in response to speech. Infants later diagnosed with (Young et al., 2009; Jones and Klin, 2013; de Klerk et al., 2014) or who screen positive for (Pineda et al., 2015) autism tend to look at others' eyes at least as much as peers in the first few months, but looking more at the eyes predicts *more* problems with face recognition (de Klerk et al., 2014), speech (Young et al., 2009; Pineda et al., 2015), and motor (Pineda et al., 2015) abilities. *Lack* of involuntary drifts of eye movement in newborns who screen positive for autism at the age of 2 years similarly predicts not only worse language but also poorer cognitive and motor development (Pineda et al., 2015). Similarly, in autistic newborns differences in functional connectivity between brain regions related to auditory–motor integration (Perani et al., 2011; Liu et al., 2019, 2020) provide an exceptionally early brain-related sign of autism (Morrel et al., 2023). Speech impairs face scanning in 6-month-old infants later diagnosed with autism (Shic et al., 2014), but at this age, attention to faces often fares in the average range but begins to decline in autistic infants as other make more of it (Jones and Klin, 2013; Rutherford et al., 2015; Gangi et al., 2021). It appears autistic infants turn away to better listen, like autistic adults (Garvey et al., 2024; for autistic children also see Doherty-Sneddon et al., 2012; Falck-Ytter, 2015).

Autistic infants also turn their attention to mouths, arguably to try to integrate speech sounds with lip motion (lip-read: Kapp, 2016), yet they show challenges with sensory integration (Zhou et al., 2025). Autistic people experience lifelong audiovisual challenges (including filtering out background noise) that affect speech perception and far beyond (Feldman et al., 2018; Callejo and Boets, 2023; Choi et al., 2023; Jertberg R. M. et al., 2024). Autistic children continue to need time to turn their attention to mouths to lip-read because of difficulties processing the speech sounds at the same time as a moving face (Newman et al., 2021; Polzer et al., 2024). Lipreading provides helpful lifelong audiovisual integration (see Kapp et al., 2019a; Newman et al., 2021).

Eye contact between caregivers and infants or children is uncommon for both autistic and non-autistic peers and is not required for joint attention between them (Adolph and West, 2022; Yurkovic-Harding et al., 2022; Thorup et al., 2024). Autistic children with hypersensitive perception alongside motor differences may exhibit enhanced covert attention: the ability to see with little eye movement (Gernsbacher et al., 2008b,c). However, the caregiver not understanding this and “seeing the world through their child's eyes” to understand their bids for joint attention, may contribute to why such children often have lower language skills (Amso et al., 2014; see Kapp, 2018b). Some autistic people acquire language (such as the ability to read and write if not speak), regardless of joint attention (Kissine et al., 2023).

#### Social responsiveness: autonomic differences in sensory perception

Autistic infants' difficulties in processing motion may increasingly (in combination with other developmental demands) challenge their ability to respond to typical social interactions. Autistic infants show differences in global motion processing associated with later language (Nyström et al., 2021; Hardiansyah et al., 2023; Hedenius et al., 2023), paralleling the lifelong challenges autistic people have with processing motion (Van der Hallen et al., 2019). Others' movement (rather than sociality *per se*) may challenge perception in autistic infants, as unusual neural response to shifts in but not static eye gaze in infants predicts autism diagnosis at the age of 3 years (Elsabbagh et al., 2012). Infant processing parallels neurological differences (Weisberg et al., 2014) and spontaneous attention (Hanley et al., 2013) in how autistic adolescents and young adults process dynamic representations of social stimuli (video clips) but not static images.

Furthermore, autistic infants show differences in global *form* processing associated with later visual cognition (Nyström et al., 2021; Hedenius et al., 2023), paralleling autistic people's capacities with processing static images and strengths with visual-spatial tasks. Enhanced visual search at 9 months predicts higher autism manifestations at 15 months and 2 years (Gliga et al., 2015), demonstrating developmental continuity with superior visual search related to more obvious autism in autistic children and adolescents (Joseph et al., 2009; Keehn and Joseph, 2016). This group difference primarily occurs for trials with absent goals (Keehn and Joseph, 2016), again suggesting differences in relatively involuntary perceptual processes. Indeed, perceptual peaks in cognition in autistic people do not necessarily relate to their interests but draw from innate processes (Meilleur et al., 2015; Courchesne et al., 2020).

Autistic infants may experience heightened arousal and perception in response to growing sensory and other demands. Greater pupil size in reaction to emotional faces in infant siblings of autistic children at 9 months predicts greater social communication differences at 18 months (Wagner et al., 2016). This finding mirrors an enhanced pupillary reflex to light in infancy, the size of which predicts their autism diagnosis and autistic traits in toddlerhood (Nyström et al., 2018). This reflex declines in the second year around the time “developmental regression” tends to occur (Fish et al., 2021; Tan et al., 2021). While dysregulated pupil dilation for sensory (or attentional) reasons may interfere with this reflex for many autistic children (de Vries et al., 2021; Zhao et al., 2022), when autistic youth show an enhanced pupillary light reflex at sensory-perceptual and attentional levels, it relates positively to social cognition performance (with the opposite pattern for non-autistic peers: Bast et al., 2019). This may suggest that enhanced perception can help autistic people perceive non-verbal cues through faces and body language when not too overstimulating. Indeed, allowing more time to process movement (Robertson et al., 2014) such as slowing down the presentation of facial expressions (Gepner et al., 2001, 2021) or offering color filters of autistic people's choice helps them read (words and people: Ludlow et al., 2006, 2012, 2020; Whitaker et al., 2016) or imitate them (Tardif et al., 2007; Lainé et al., 2011).

The auditory modality further demonstrates these basic perceptual processes. It illustrates support for classic theory on autism as arising from physiological overarousal (Hutt et al., 1964) and sensory and motor differences related to the vestibular function of the brainstem (Rimland, 1964; Ornitz, 1974; Ornitz et al., 1985), which continue to accumulate evidence (Delafield-Butt and Trevarthen, 2017; Dadalko and Travers, 2018; Burstein and Geva, 2021). Among the evidence of autism-related auditory hypersensitivity in infancy, only for those with elevated auditory brainstem response as newborns did a higher preference for stimulation at 4 months predicting signs of autism (especially social ones) and difficulty with speech at the age of 3 years. These findings across the three time points predicted an autism diagnosis and more obvious autism (Cohen et al., 2013). Those results parallel prolonged, narrow auditory brainstem responses in autistic newborns (Miron et al., 2016; Torres et al., 2023), and similarly slow responses in autistic children (Rosenhall et al., 2003). They also dovetail with evidence of expanded windows of time for multisensory perception in autistic people (Foss-Feig et al., 2010; Baum et al., 2015) and broader challenges with the timing of processes within autistic people and between them and communication partners (Murat Baldwin et al., 2022).

While attention plays a role in perception, evidence suggests bottom-up processing grounded in the surrounding sensory (and social) environment occurs through relatively automatic processes not necessarily driven by interests (as much as they may add joy to life). An earlier adaptation (Kapp, 2013) of Donnellan et al.'s (2010, 2013) sensory–movement account suggested the importance of the salience network, which integrates external sensory stimuli with one's own bodily, emotional, and mental states (Uddin et al., 2013). Hyperconnectivity in this network in 6-week-old babies recently predicted autism-related behaviors, and greater connectivity with sensory regions related to parent-reported sensory behaviors, within the first year (Tsang et al., 2024). This parallels brain patterns in autistic children (Green S. et al., 2016).

Studies like those suggest greater attention to sensory stimuli (because of distraction, pleasure, or pain) and indeed lack of attentional disengagement may stem from sensory sensitivities beyond eye-movement difficulties. For autistic infants, larger pupil dilation when listening to a nonsocial sound (running water) predicted autism diagnosis and related to “stickier attention” (less shift in gaze), whereas their response to baby-talk did not differ from “comparison groups' response” (Rudling et al., 2022). Various autism-typical features such as a strength in resistance to distraction or difficulty at the level of eye movement control may contribute to hyperfocus in some infants (c.f. Gernsbacher et al., 2008c). A lifelong lack of attentional disengagement often occurs for some autistic people (Keehn et al., 2013; Sacrey et al., 2014a), related to greater hypersensitivity and sensory seeking (Sabatos-DeVito et al., 2016). It also may relate to poor oculomotor control that contributes to language-impaired status among autistic people (Kelly et al., 2013) and may relate to more obvious autism (Ziv et al., 2024). Furthermore, co-occurring ADHD may contribute to difficulties controlling attention (Dupuis et al., 2022).

#### Interests

For all these reasons and more, autistic people and their communication partners may struggle to understand or interact with one another (particularly those more dissimilar from themselves, Milton, 2012). As described by the autistic-designed monotropism account, when this happens young autistic people might withdraw to immerse in other interests (Murray et al., 2005). Parent memoirs that describe children they perceived as typically developing “regress into” autism shared successes with helping the autistic child immerse in their interests and use them as a learning opportunity (Isaacson, 2009; Barnett, 2013; Suskind, 2014). One might consider encouraging their child's “spark” (passionate interests: Barnett, 2013) a good developmental principle rather than “affinity therapy” (Suskind, 2014), as they can support learning words (Arunachalam et al., 2024) and promote flourishing in education (American Psychiatric Association, 2013; Gunn and Delafield-Butt, 2016), communication and interaction or play (Gunn and Delafield-Butt, 2016; Harrop et al., 2019), and employment (American Psychiatric Association, 2013).

Autistic people's task immersion may also stem in part from movement differences of getting relatively “stuck” in action. It has elements of both strengths in hyperfocus or resistance to distraction (e.g., from goal-based or interest-driven attention: Dupuis et al., 2022; Gernsbacher et al., 2008c) as well as difficulties in initiation and disengagement (Hill and Bird, 2006; Buckle et al., 2021), or a mixture (Russell et al., 2019; Dwyer et al., 2024; Heasman et al., 2024; Rapaport et al., 2024a,b). This area forms part of the sensory–movement account, which also emphasizes the role stress can play (Donnellan et al., 2006).

## Bottom-up processing: reduced perceptual narrowing

Autistic people's brains seem to process repeatedly encountered sensory stimuli as relatively “new”. This may relate to both unimodal perceptual strengths (e.g., in the visual and auditory modalities) and reduced integration (Heffler and Oestreicher, 2016). It may form part of a larger pattern of reduced perceptual narrowing in autistic people (Hadad and Yashar, 2022), which requires multisensory integration for frequently encountered stimuli (Lewkowicz and Ghazanfar, 2009; Maurer and Werker, 2014; Schwarzer, 2014). Instead of perceptual narrowing becoming accelerated late in the first year (Lewkowicz, 2014), autistic infants' differences with processing more of their surroundings as uncertain may overwhelm their senses and distress them, resulting in challenges in self-regulation and reduced social approach (Garon et al., 2016).

Autistic people's brains show evidence of compensation while they take in more sensory information. Neural hyperconnectivity at approximately 6 months of age that precedes reduced brain connections in those areas and autism diagnosis (Wolff et al., 2012, 2015; also see Liloia et al., 2024), may indicate transitory neural compensation as the amount of information becomes overwhelming. Similarly, autistic infants show unusually accelerated brain growth in the cerebrum around the time (6 months and especially 1 year) autism starts to become more noticeable (Dawson et al., 2023), and larger brain size in relation to apparent developmental regression (Nordahl et al., 2011). Early accelerated growth of the amygdala (Dawson et al., 2023), the novelty and emotional salience center of the brain, further aligns with the notion that autistic infants (like older autistic people) become less habituated to prior experiences that perceptual narrowing facilitates. Reduced sensory (e.g., visual and auditory) habituation in autistic people relates to more obvious autistic traits (Jamal et al., 2021). Reduced sensory habituation occurs more in autistic than in other neurodivergent people (Merchie and Gomot, 2023).

Reduced perceptual narrowing relates to theory and evidence of autism as involving strengths in bottom-up processing (grounded in the surrounding sensory experiences) or disincline toward or difficulties with top-down processing (filtered by prior experiences and expectations). Historic accounts of autism and more recent evidence suggest autistic toddlers and children may perceive physical details in the environment peers and adults do not notice (Kanner, 1943; Asperger, 1944, 1991; Losh and Capps, 2006; Klin et al., 2009). Autistic people have reduced susceptibility to a variety of optical illusions (Simmons et al., 2009), most directly “inattention blindness” of failing to see something in their visual field (Swettenham et al., 2014). This relates to visual perceptual strengths that distinguish them from others with realistic visual processing (Bölte et al., 2007). Similarly, autistic adults tend to show a visual perceptual style of processing the environment on a case-by-case basis (Johnson et al., 2010; Yechiam et al., 2010; Solomon et al., 2015), and sharper performance for nearby stimuli related to the degree of autistic traits (Robertson et al., 2013). Such tendency toward visual details does not necessarily impair holistic processing, but integration may require conscious attention (Happé and Frith, 2006; Mottron et al., 2006; Koldewyn et al., 2013), time (Van der Hallen et al., 2015) or the presence of global and local cues (Johnson et al., 2010). Realistic visual processing and visual hypersensitivity to complex motion do continue to connect to difficulties with movement (including imitation) and autism manifestations across age and intellectual abilities (Freitag et al., 2008; Price et al., 2012; Cook et al., 2013), challenging the perception of inconsistent stimuli whether of objects or people. As related again to eye contact and face processing (but extending far beyond), autistic children, adolescents, and adults process faces over a large window of time (Stevenson et al., 2016), including reduced habituation in the amygdala (Kleinhans et al., 2009; Swartz et al., 2013; Wiggins et al., 2014; Green et al., 2019). The latter means less calming over time despite repeated exposure, which suggests processing familiar faces as “new” and intense, as supported by studies that include eye-tracking (Dalton et al., 2005; Tottenham et al., 2014; Stuart et al., 2023).

Paradoxically, a bottom-up processing account of autism predicts that sometimes *non-*autistic people have more resistance to change. Autistic infants may exhibit reduced habituation to frequently heard sounds (Guiraud et al., 2011), which would suggest processing repeatedly encountered words as relatively new and an advantage when presented with a sound outside the pattern. Indeed, 1-year-old infants later diagnosed with autism showed a greater understanding and production of unexpected words when controlling for their lower language skills (Lazenby et al., 2016). This parallels how autistic children's performance when learning a new movement showed less adverse impact than typically developing peers and those with ADHD when presented with an alternative pattern (Gidley Larson and Mostofsky, 2008). Indeed, autistic adults recently performed better in an *un*predictable task compared with non-autistic people, contrary to researchers' expectations (Lacroix et al., 2025). Similarly, although autistic adults self-reported lower intuition, they responded *more* intuitively (Bastan et al., 2024). Research generally finds that autistic people rely more on new evidence than prior experiences when making decisions (Van der Plas et al., 2023), which may enable them to “jump to conclusions” more so than non-autistic peers (Sahuquillo-Leal et al., 2020).

## Integrative neuroscience: perception–action decoupling and feedback loops

Such bottom-up processing may itself grow from lifelong reduced sensory integration in feedback loops involving the cerebellum of the brain. Among brain regions, the cerebellum may particularly illuminate autism as a permanent neurodivergence, as it has the most neurons (Fiez and Stoodley, 2024) and evidence for a lifelong (smaller) pattern (Liloia et al., 2024), especially through direct testing in post-mortem studies (Fetit et al., 2021). Therefore, it may best substantiate autistic people's tendency (unlike non-autistic people) to think that only biology causes autism (Kapp et al., 2013). Belief in autism's innateness and neurobiological “hard-wiring” relates to the rejection of the possibility of curing autism (VanDaalen et al., 2025). While this view might reflect biological essentialism (Russell, 2020), growing evidence shows that neurological differences in infancy predict meeting diagnostic criteria for autism (Ayoub et al., 2022; Dawson et al., 2023; Morrel et al., 2023). Those autistic people who manage to engage in tasks typically difficult for autistic people like understanding figurative language (Herringshaw et al., 2016), or apparently no longer meeting current behavioral criteria for autism (Eigsti et al., 2016), may engage more of their brain across hemispheres to do so compared with non-autistic people and other autistic people. This may suggest compensation (or camouflaging) rather than normalization (see Eigsti et al., 2016). Indeed, the brain activity of autistic adolescents and adults who may not necessarily still *appear* autistic relates to their behavior in early childhood rather than the present (Ecker et al., 2012, 2013); such camouflaging contributed to the allowance of diagnosis of autism by history (American Psychiatric Association, 2013; Kapp and Ne'eman, 2020).

Thus, the cerebellum may help illustrate the relative stability of the underlying autism even as expressed skills may fluctuate. A researcher focused on “regression” into autism emphasized the cerebellum for multisensory feedback loops (Kern, 2002), as does the much different sensory–movement account of autism (Donnellan et al., 2010). The region integrates signals between other brain regions and the sensory systems of the spinal cord for muscular activity, and evidence continues to grow for its roles in perception and social cognition beyond sensorimotor learning and control in autistic people (Rice and Stoodley, 2021; Biswas et al., 2024) and in the general population (Olson et al., 2023; Fiez and Stoodley, 2024).

A smaller cerebellum predicts autism in newborns (Ure et al., 2016) and shows exceptional lifespan stability in lobules VI-VII (Courchesne et al., 2011; Crucitti et al., 2020; Liloia et al., 2024; Mohammad et al., 2024a but see Sussman et al., 2015; Laidi et al., 2022; Klaus et al., 2024). This reduced size relates to the decoupling of perception and action for motor learning (Marko et al., 2015) and reduced motor memory (Neely et al., 2019). It relates to the visibility of autistic traits across domains (D'Mello et al., 2015), such as reduced exploration of the environment alongside greater stimming (Pierce and Courchesne, 2001). A smaller cerebellum applies especially to autistic people with lower daily living skills (Duan et al., 2024), higher support needs (Riva et al., 2013), and a history of speech onset divergence (Kevin et al., 2011) even when matched for IQ as children (D'Mello et al., 2016) and adults (Lai et al., 2015). It distinguishes autism from ADHD and dyslexia (associated with larger volume: Stoodley, 2014) and inversely also intellectual disability (Kaufmann et al., 2003), developmental delay (Webb et al., 2009), and developmental language disorder (Hodge et al., 2010).

The cerebellum in autistics has also shown patterns of high and low connectivity with other brain regions (Stoodley, 2014; Hanaie et al., 2018), which may help classify autism (Yi et al., 2024). These pathways in newborns predict autism, motor, and language challenges (Cook et al., 2023); in autistic infants, they predict sensory responsiveness (Wolff et al., 2017) and social manifestations (Okada et al., 2022). The nerve tracts of the cerebellum in autistic people distinguished it from dyspraxia, related to greater social differences (Kilroy et al., 2022a). In autistic people, the cerebellum has exhibited enhanced connectivity with sensory and motor networks (Khan et al., 2015b; Oldehinkel et al., 2019; Wang et al., 2019) that grows over time (Lepping et al., 2022). It also shows reduced connectivity with error monitoring, socioemotional, or language regions (Verly et al., 2014; Arnold Anteraper et al., 2019; Wang et al., 2019; Unruh et al., 2023), such as related to social cognition (Igelström et al., 2017) or attention to others' eyes (Laidi et al., 2022). Hyperreactivity to sensory stimuli and involuntary attention explain these cerebellar connectivity patterns that relate to greater manifestations of autism (Lidstone et al., 2021; Alho et al., 2023; Cakar et al., 2024).

The cerebellum also helps account for gender differences among autistic people. It shows unique gender structural differences within different subregions (Supekar and Menon, 2015), with smaller size related to more obvious autism for males and more RRBIs for females (McKinney et al., 2022), and unusual changes in size over time that differ by gender (Sussman et al., 2015). Greater cerebellar activity in autism-associated visual-spatial strengths has been studied more with males (Thérien et al., 2022, 2023). Among males, autistic people uniquely showed higher cerebellar activity during visual-spatial tasks (Manjaly et al., 2007), including as related to faster response times (Thérien et al., 2023; also see Hayes et al., 2018) and higher visual-spatial complexity (Thérien et al., 2022). Nevertheless, opposite patterns of cortical–cerebellar connectivity in autistic females (high) and males (low) may help explain why some studies have failed to find unusual connectivity in mixed-gender samples of autistic people (Smith et al., 2019; Linke et al., 2020; Gaudfernau et al., 2023; Hawks et al., 2023). This might result from greater social camouflaging in autistic females despite their greater sensitivities (Lai et al., 2011; Eigsti et al., 2016).

Contrasting patterns for the connectivity or size of the cerebellum in autistic people who differ by possessing (or not) common co-occurring neurodivergences such as ADHD (Surgent et al., 2024) and depression (Dhar et al., 2024) suggest replications are needed for how the region helps “get a grip” on diversity among autistic people. Furthermore, autistic people's sensory responsiveness may also relate well to other regions such as the brainstem (Surgent et al., 2022), suggesting the need for a whole-brain approach.

## Grasping the implications: consequences of sensory differences and social contexts

Sensory and movement differences impact autistic people's daily lives, with often heightened recognition by autistic people. Autistic children have long identified movement-relevant challenges such as low athleticism alongside social differences (Capps et al., 1995; Bauminger et al., 2004; Williamson et al., 2008). Autistic young people and adults have similarly long reported worse or equal physical quality of life than their parents reported, with other domains such as social relationships in the opposite direction (Shipman et al., 2011; Sheldrick et al., 2012; Hong et al., 2016). To the extent that autistic traits impact quality of life (Kim and Bottema-Beutel, 2019), they may impact school achievement and physical health (Oakley et al., 2021). Reactivity to sensory stimuli may have this consequence, as it challenges classroom learning (especially in the auditory domain: Ashburner et al., 2008; Howe and Stagg, 2016), and uniquely predicts both poor physical and psychological health (Lin and Huang, 2019). For example, hypersensitivity challenges supermarket visits (MacLennan et al., 2023), comfortable clothing (Kyriacou et al., 2023), healthy eating (Nimbley, 2024), dental care (McMillion et al., 2021; Chauhan et al., 2024), menstruation (Steward et al., 2020; Fearon et al., 2025), sex (Barnett and Maticka-Tyndale, 2015), childbirth (Gardner et al., 2016), and menopause (Moseley et al., 2020; Karavidas and de Visser, 2022; Brady et al., 2024). Autistic people (especially those assigned female at birth) with less expressive language may struggle to have their needs met and become so physically overwhelmed with pubertal changes that their bodies may at times take hold of them, possibly helping explain the unusual onset in adolescence of epileptic seizures (Rutter and Pickles, 2016) and catatonic motor freezes (Dell'Osso et al., 2022) disproportionately affecting them. Sensory overload may result in uncontrollable meltdowns (Lewis and Stevens, 2023). While arranging environments and routines may reduce overwhelm (Daly et al., 2022), even “autistic space” cannot always resolve competing sensory access needs (Sinclair, 2010).

### Moving (further) toward sensitive understanding of autism: underestimated empathy and pain in autistic people

It appears that people have internalized views about autism such that autistic people and their families may endorse stereotypes even where the evidence points to the contrary. Ongoing (mis)conceptions of empathy in autistic people may lead to its under-recognition, even as sensory overstimulation in non-autistic people is long established to positively relate to empathy (Aron and Aron, 1997; Gubler et al., 2024). Autistic people's hypersensitivities significantly overlap with this “highly sensitive person” (HSP) construct, helping account for their tendencies toward introversion and vulnerability to stress (Schwartzman et al., 2015), yet the author may have sought to avoid making an HSP–autism association because of the stigma of diagnoses (Edenroth-Cato and Sjöblom, 2022). Parents tend to under-report their autistic children's demonstrated empathetic responsiveness, in association with more obvious autism (Scheeren et al., 2013). Similarly, autistic adults tend to under-report their own empathy compared with their tested empathic response (Trimmer et al., 2017), although many autistic people report high empathy (Kimber et al., 2024). Similarly, families may under-recognize sensitivity to pain, which forms part of tactile or somatosensory processing (Moore, 2015; Zhang et al., 2021). Parents under-report their autistic children's pain (more so than parents of non-autistic children) even when their children have observable facial expressions displaying it (Nader et al., 2004). Again similarly, autistic youth may under-recognize or under-report their own pain (Duerden et al., 2015).

## Moving forward: testing the theory and applications to support for autistic people

This theoretical model raises questions about whether the autism diagnosis adequately explicitly covers sensory and in particular movement (and related) differences when they so often form such a central role in autistic people's experience of autism; arguably, motor challenges merit addition to the diagnostic criteria (Miller et al., 2024). Meanwhile, this account raises concerns about the dangerousness of placing social dysfunction within single people. It also raises issues with the limitations of a behavior-based diagnosis despite the lack of reliable biomarkers (Loth, 2023). It is limited by the scarcity of research on the perspectives of autistic people with intellectual disability (Gibbs et al., 2024). Further participatory research with diverse autistic community members and researchers on autism as a construct and diagnosis would help (Ratto et al., 2023). It also could strengthen measurements and approaches to sensory processing (Nicolaidis et al., 2020; Gunderson et al., 2023; Williams et al., 2024; He et al., 2023), which lacks good measurement even for non-autistic people (Greven et al., 2024).

The theory of autism as a lifelong sensory–movement neurodivergence is testable through prospective longitudinal research on whether sensory, motor, and domain-general differences continue to appear as the earliest signs of autism, especially neurologically from birth. For example, studies may focus on neurological differences in autistic *newborns* in the cerebellum, such as whether it relates to (a) differences in learning and movement during sleep that predict an autism diagnosis (see Fifer et al., 2010; Denisova and Wolpert, 2024), (b) within-person differences in sensation and movement in autistic people via “idiosyncratic” network connections (see Vakorin et al., 2017), and (c) differences in perceiving (social) action that predict challenges learning higher-order rules like the social “hidden curriculum” (see Balsters et al., 2013; Olson et al., 2023; Myles et al., 2024). Other relevant regions include the somatosensory cortex involved in touch and proprioception, which showed particular potential for distinguishing between autistic and non-autistic children's and adult's brains at rest (Chen et al., 2015). Further fine-grained research on early developmental regression in autistic people may try to distinguish between losing and not showing skills consistent with nuances in “older” autistic children and adults, for example, whether young children who seem to lose access to their speech may still express inner speech (see Alderson-Day and Pearson, 2023). Selective mutism (Muris and Ollendick, 2021) may apply to apparent lost speech or language, which may have prolonged episodes if children struggle to communicate or do not feel comfortable speaking. Early-arising autistic burnout (Raymaker et al., 2020)—if not autistic inertia (difficulties with starting and stopping consistent with the sensory–movement account: Buckle et al., 2021; Donnellan et al., 2006) or even catatonia (Smith et al., 2025)—may account for more general declines (Baggs, 2005).

Longitudinal research might investigate the relationship between “developmental regression” and these forms of pause or regression usually more associated with later development. For example, a measure of autistic traits in adulthood co-produced with autistic people defines within-person variability and the at least temporary loss of skills as part of autism (Ratto et al., 2023). Similarly, after high school or by their 30′s, many autistic people experience declines in daily living skills (Smith et al., 2012; Clarke et al., 2021; Tomaszewski et al., 2025), to which the reduced sensory tolerance in autistic burnout may contribute (Raymaker et al., 2020).

The extent to which subtle early differences reflect a form of “camouflaging” merits further study (Petrolini et al., 2023). Autistic people's brain differences that precede more obvious behavioral differences draw some parallels to people who may no longer *appear* to meet diagnostic criteria still showing autism-related neurological (Eigsti et al., 2016), cognitive, and experiential (Lai et al., 2011) differences. They may still actively repress hand-flapping (Padawer, 2014) and have distressing sensory differences (Thomas, 2023). This has practical relevance because they are still visibly different (Canale et al., 2024), and subtler autism relates to more risk of victimization (Kapp, 2018b; Libster et al., 2022), especially as they show poor social judgment alongside extreme friendliness and approachability (Orinstein et al., 2015). These phenomena may help explain why autistic people whose diagnoses are removed may not be happier (Pickles et al., 2020).

### Sensory-sensitive support for autistic people in and beyond interactions

Sensory and movement difficulties have implications for supporting young autistic children, including slowing down body language (Lainé et al., 2011; Gepner et al., 2022), and giving them color filters that help them read words and people (Ludlow et al., 2012, 2020; Whitaker et al., 2016); encouraging AAC and language (Almirall et al., 2016; Logan et al., 2017), lip-reading (Kapp et al., 2019a; Habayeb et al., 2021; Newman et al., 2021), interactive stimming (Sinclair, 2005; Chen, 2024; Morris et al., 2025); imitating their movements (Nadel, 2015), and taking a more responsive than directive approach when interacting (Kapp, 2018b). However, speech (Chazin et al., 2024; Schuck et al., 2024) and eye contact (Stuart et al., 2023; Garvey et al., 2024) should not necessarily be encouraged.

Future research may explain whether tactile hypersensitivity mediates (or at least moderates) the impacts of developmental interventions adapted from positive parenting programs that encourage parents to follow their child's lead. Tactile processing develops first among sensory systems and differences in it relate to various neurodivergences (Cascio, 2010), with exceptional (among the traditional five modalities) near-universal frequency in young infant siblings of autistic children (Van Etten et al., 2017). Aversion to social touch, whether naturally in the home (Baranek, 1999) or experimentally tested by parents (Mammen et al., 2015), in infants late in the first year predicts autism manifestations and diagnosis. Conversely, seeking touch in infancy reduced the likelihood of showing signs of autism in toddlers, even among infants with reduced neural habituation to tactile input (Piccardi et al., 2021; also see Narvekar et al., 2024). Reduced parental affectionate touch toward autistic infants in response to crying (Esposito and Venuti, 2009) or in interaction (Apicella et al., 2013) may reflect an adaptation, as tactile-hypersensitive autistic children show a defensive behavioral reaction to touch, regardless of whether a person does the touching (Cascio et al., 2016; Riquelme et al., 2016). Quality, reliable relationships may benefit tactile-hypersensitive autistic people (Robledo and Donnellan, 2008, 2016), as autistic people struggle with unexpected touch (whether or not it is social: Cascio et al., 2015; Duerden et al., 2015; Khan et al., 2015a). The particular benefit (vs. comparison peers) of responsive as opposed to directive parenting on positive social engagement in infant siblings of autistic children (Harker et al., 2016) and on language development in autistic toddlers (Baker et al., 2010), across relationship context (parent, teacher, or therapist: Goods et al., 2013; Mohammadzaheri et al., 2014b; Patterson et al., 2014; Pellecchia et al., 2015), may in part reflect less intrusive touch. Indeed, hyper-responsiveness to touch mediated social inhibition and more struggles in groups among institutionalized autistic adults with intellectual disability (Lundqvist, 2015).

Different beliefs about autistic people's development have historically caused anguish among families of autistic people, and threaten to repeat harm if caregivers think of autism as foreign to a child (Broderick and Ne'eman, 2008; Sarrett, 2011). The false belief that cold “refrigerator mothers” cause autism destroyed families and put autistic people in institutions (Kapp, 2022). Ironically, children raised in deprived orphanages (Rutter and Sonuga-Barke, 2010) or adopted after caregiver maltreatment (Green J. et al., 2016) are much more likely to meet behavioral criteria for autism, although leaders in the autism field have described this as “quasi-autism” because of this history and belief in autism as relatively organic (Rutter and Pickles, 2016). Many of those children may have environmentally induced tactile defensiveness from social deprivation (a lack of reliable, calming touch), but their signs of autism decline when adopted into a loving home (Rutter and Pickles, 2016). Similarly, congenitally blind people disproportionately meet diagnostic criteria for autism but many have unusual or subtler presentations that often substantially disappear over time (Hobson and Lee, 2010; Jure et al., 2016), perhaps reflecting learning effective tactile processing through relying on it as blind people often do (Legge et al., 2008; Wan et al., 2010). Autistic children's innate tactile differences may resemble these other neurological or environmental states, but their different cause and function arguably require accommodation.

With the rise of extremely early interventions based on positive parenting models before infants show many signs of autism, the autism field risks returning to a “quasi-refrigerator mother” era (see Kapp, 2018a). These studies have found little impact (McGlade et al., 2023), despite a headline-grabbing study that falsely implied autism could be prevented (Whitehouse et al., 2021; see Dwyer et al., 2021). Many autistic people and parents do not want these or later interventions to try to reduce autistic traits or apply certain behavioral approaches (Bent et al., 2024; Sulek et al., 2024), although many parents do see them as beneficial even for non-autistic children (Washington et al., 2024) and wish for genetic testing because of stigma (Fischbach et al., 2016).

The neurodiversity movement tries to help parents unnecessarily guilt-ridden about the cause of their child's autism accept and support their children to leverage their autism positively and authentically (Kapp, 2022). Its co-founding leader Jim Sinclair encouraged parents to learn their child's language (Sinclair, 1993; Pripas-Kapit, 2020), which has near-universal support in the autism community (Kapp et al., 2013). Autistic people support early diagnosis *if* it helps parents accept their child (Vanaken, 2023; Riccio et al., 2021). Certainly, parental acceptance of the autism diagnosis and seeing things from the child's perspective helps parents engage with and relate to their child (Kapp, 2018b; Di Renzo et al., 2020). Furthermore, caregivers of autistic infants may not show reduced responsiveness as a group (Wan et al., 2019). They are more likely to be responsive to infants with greater sensory and movement differences (Campi et al., 2024; Ke et al., 2025), perhaps because they recognize signals from sensory hyperresponsiveness.

### Applications across autistic people (e.g., non-speaking, multiply neurodivergent, majority world)

While autistic people form a diverse group, this account works best for people who meet all four RRBI criteria of the current autism diagnosis (as well as all three social communication criteria as standard), who likely have sensorimotor differences (Miller et al., 2014; Lord et al., 2015). For autism to be a valid and cohesive construct, its total main components need to meaningfully interrelate for some people. This model also likely works better for autistic people with speech divergence^1^, who the sensory–movement account has prioritized (Kapp, 2024). Autistic people with genetic syndromes and often co-occurring intellectual disabilities may appear to show less hyperresponsiveness (Hudac et al., 2024), but whether this reflects shutdowns, inertia, or catatonia merits further investigation. The theory applies to multiply neurodivergent autistic people. Autistic people with ADHD may have more sensory differences than autistic people without ADHD, with autism explaining enhanced perception, sensory hyperresponsiveness, and worse motor skills (Skaletski et al., 2024; also see Surgent et al., 2024). Autistic people with and without cerebral palsy overlap in showing patterns for muscular hypotonia yet relatively strong ambulatory skills (Ming et al., 2007; Kirby et al., 2011; Christensen et al., 2014; Bishop et al., 2016). Deaf autistic people overlap with hearing autistic people in showing visual strengths (Maljaars et al., 2011) and possible fine motor weaknesses (Seal and Bonvillian, 1997; Shield and Meier, 2012), alongside underestimated social cognitive competencies (Shield et al., 2016).

Furthermore, the details in these studies reflect a synthesis of mostly Western, Educated, Industrialized, Rich, and Democratic (WEIRD) societies (Henrich et al., 2010), reflecting both the state of the autism field and the neurodiversity movement establishment (Nair et al., 2024). Developmental “milestones” and social norms are culturally relative even for motor development (Karasik and Robinson, 2022), and children in societies with strikingly different practices may acquire motor skills and experiences at different times while resulting in similar functioning (Karasik et al., 2023).

## Toward understanding, accepting, and supporting autistic people

While no theory can fully explain autism (Happé et al., 2006), this paper has attempted to synthesize how sensory–movement differences may help provide evidence for autism as a lifelong neurodivergence rather than a social communication disorder, even as its manifestations may dramatically change over time. While deep engagement with this literature exceeds the scope of the present paper, the theory is consistent with autistic people not only having an interest in relationships but also more inherent struggles such as sensory overload and misunderstandings from others (especially non-autistic people), which at times challenge engaging with them (Kapp, 2013; Murray et al., 2023). Further understanding (especially neuroscientifically) may increase our ability to provide the recognition and support autistic people need.

## Data Availability

The original contributions presented in the study are included in the article, further inquiries can be directed to the corresponding author.

## Footnotes

^1^Kapp, S. K., and Gudknecht, J. (Under review). *The Strengths of Speech Divergence in Autistic People: Implications for Assessment and Support*.
